# Directed Evolution Detects Supernumerary Centric Chromosomes Conferring Resistance to Azoles in Candida auris

**DOI:** 10.1128/mbio.03052-22

**Published:** 2022-11-29

**Authors:** Aswathy Narayanan, Praveen Kumar, Anshu Chauhan, Mohit Kumar, Kusum Yadav, Atanu Banerjee, Ravi Datta Sharma, Shivaprakash M. Rudramurthy, Arunaloke Chakrabarti, Kaustuv Sanyal, Rajendra Prasad

**Affiliations:** a Molecular Mycology Laboratory, Molecular Biology and Genetics Unit, Jawaharlal Nehru Centre for Advanced Scientific Research, Bangalore, Karnataka, India; b Amity Institute of Integrative Science and Health, Amity University Gurgaon, Gurgaon, Haryana, India; c Postgraduate Institute of Medical Education a Research, Chandigarh, India; d Osaka University, Osaka, Suita, Japan; Yonsei University

**Keywords:** drug resistance, experimental evolution, extra chromosomes, karyotype

## Abstract

Candida auris exhibits resistance to multiple antifungal drug classes and sterilization agents, posing threats to the immunocompromised worldwide. Among the four major geographical clades, the East Asian clade 2 isolates of C. auris are mostly drug susceptible. In this study, we experimentally evolved one such drug-susceptible isolate for multiple generations in the presence of the antifungal compound fluconazole and analyzed changes in the karyotype, DNA sequence, and gene expression profiles in three evolved drug-resistant isolates. Next-generation sequencing and electrophoretic karyotyping confirm the presence of segmental aneuploidy as supernumerary chromosomes originating from centromere-inclusive chromosomal duplication events in two such cases. A 638-kb region and a 675-kb region, both of which originated from chromosome 5 and contained its centromere region, are instances of supernumerary chromosome formation identified in two evolved fluconazole-resistant isolates. Loss of the supernumerary chromosomes from the drug-resistant isolates results in a complete reversal of fluconazole susceptibility. Transcriptome analysis of the third isolate identified overexpression of drug efflux pumps as a possible non-aneuploidy-driven mechanism of drug resistance. Together, this study reveals how both aneuploidy-driven and aneuploidy-independent mechanisms may operate in parallel in an evolving population of C. auris in the presence of an antifungal drug, in spite of starting from the same strain grown under similar conditions, to attain various levels of fluconazole resistance.

## INTRODUCTION

Rising antifungal resistance has contributed significantly to the global mortality rates associated with fungal infections and the failure of fungal pathogen control in agriculture, posing threats to human health and food security (1). Response to antifungal compounds can vary at the species level. Some species exhibit intrinsic resistance to a compound, such as the azole resistance in Aspergillus fumigatus. While many fungal species are drug susceptible, various changes at the cellular level may result in emergence of a few cells in a population as drug-tolerant or drug-resistant cells like persister cells in biofilms (2). The potential of acquiring drug resistance through adaptive mutations is reflected in the evolvability of a fungal pathogen. Evolvability is the ability of an organism to produce heritable, adaptive phenotypic changes, driven by preexisting genetic variation or new mutations in the population, facilitating the process of natural selection in a specific environment (3). The evolvability of different fungal pathogens to acquire antifungal resistance has been studied previously using experimental evolution approaches. Such approaches successfully uncovered mechanisms underlying the emergence of antifungal resistance. For instance, acquired fluconazole and anidulafungin resistance in Candida glabrata developed in an experimental evolution study was attributed to a high diversity of mutations occurring in a restricted set of genes (4). Experimental evolution strategies have also revealed multiple mechanisms resulting in drug resistance in the same species. In C. albicans, overexpression of genes such as *CDR1*, *CDR2*, *ERG11*, and *MDR1* can result in azole resistance (5), while rapid acquisition of fluconazole resistance was also mediated by extensive copy number variations that amplified genes associated with drug resistance (6).

A relatively recent addition to the panel of multidrug-resistant fungal pathogens is Candida auris, isolated from an ear infection in Japan in 2009, as a drug-susceptible pathogen causing localized infections (7). It surfaced simultaneously in multiple geographical zones and later was classified as geographical clades. While clade 2 isolates are largely susceptible to antifungal drugs, all three other major clades of C. auris exhibit resistance to one or more drugs (8). Currently, C. auris has been reported in more than 30 countries across the globe as a multidrug-resistant pathogen of significant concern. Approximately 90% of the clinical C. auris isolates are resistant to the clinically common antifungal fluconazole (FLC), and >30% display increased resistance to the polyene amphotericin B (AmB) (9). Echinocandin resistance is shown by 2 to 7% of the isolates known so far (10).

Studies investigating drug resistance mechanisms in C. auris have employed drug-resistant clinical isolates to identify changes at the sequence and transcription levels. Drug-susceptible isolates have also been subjected to experimental evolution under short- or long-term drug stress to track the molecular events culminating in drug resistance. Independent studies have identified multiple aneuploidy-independent mechanisms, such as mutations in transcription factors such as *TAC1* and *UPC2*, overexpression of efflux pumps like *CDR1*, and mutations in genes involved in ergosterol biosynthesis responsible for azole resistance (11–14).

Ploidy plasticity is a proven means to adapt to stress conditions in fungi (15). C. albicans was shown to tolerate aneuploidy of each of its chromosomes that, under specific conditions, conferred a fitness advantage (16). For instance, chr2 trisomy conferred resistance to hydroxyurea as well as caspofungin (17). Disomy of chr1 has been associated with an increase in FLC resistance in Cryptococcus neoformans (18). *In vitro* studies in C. auris have also unveiled the role of aneuploidy in acquired azole resistance in the form of segmental duplications, coexisting with aneuploidy-independent mechanisms in an evolving population derived from a single parent strain (11). However, the mechanism of generation of a stable aneuploid state in C. auris remains an enigma. In the present study, we subject a drug-susceptible clinical isolate of C. auris belonging to clade 2 to *in vitro* long-term evolution in the presence of FLC to obtain drug-resistant strains. We demonstrate generation of a mitotically stable supernumerary chromosome by segmental duplication of a native chromosome, including its centromere as a novel mechanism of conferring drug resistance by increasing the copy number of a set of genes in C. auris.

## RESULTS

### Experimental evolution reveals the potential of C. auris clade 2 type strain to acquire fluconazole resistance.

**FIG 1:**
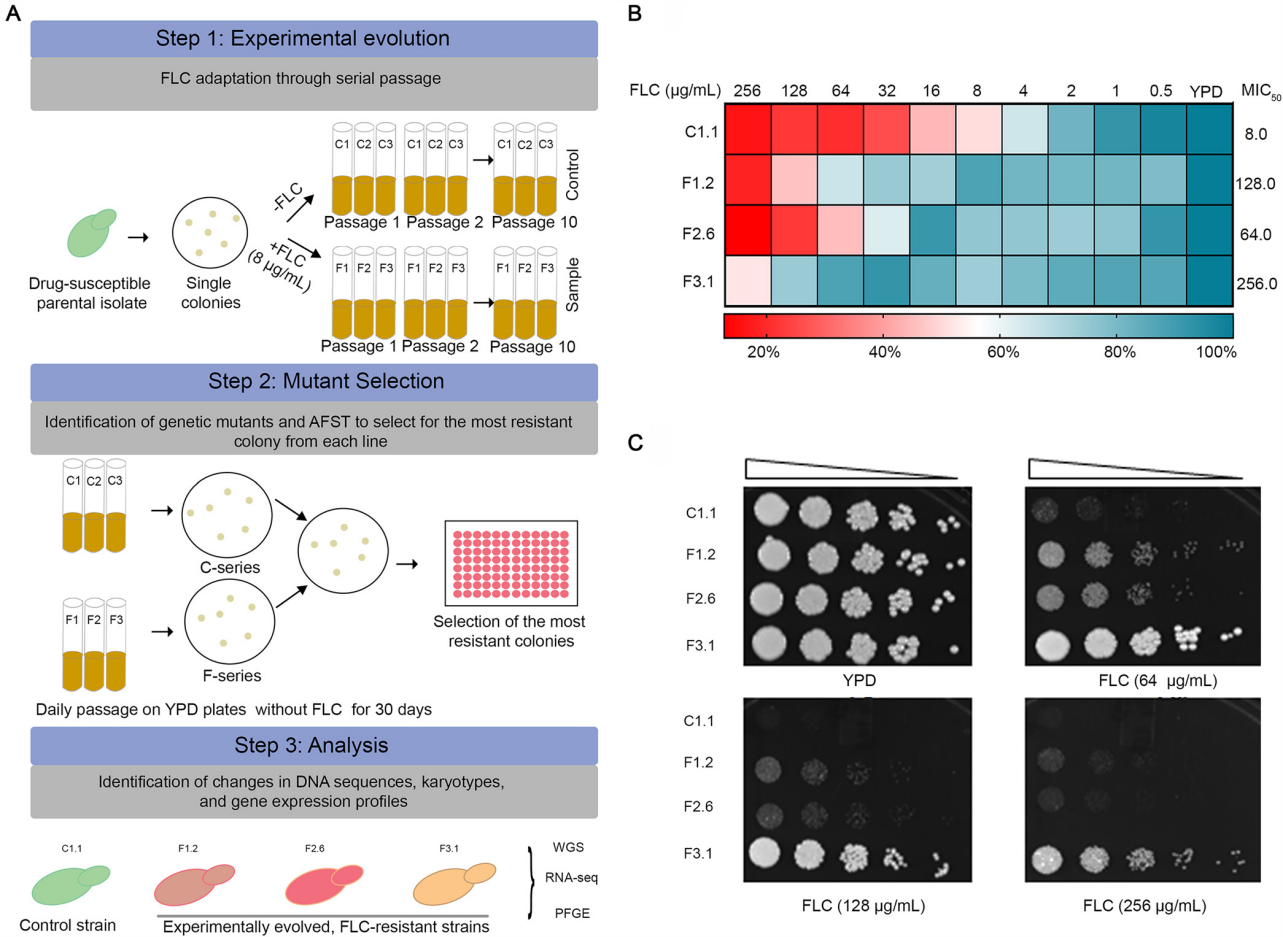
Directed evolution regime reveals evolvability of a C. auris strain toward FLC resistance. (A) Schematic of the experimental evolution of an azole-susceptible C. auris clade 2 strain. Step 1, an evolution regimen of passaging a single colony with or without FLC. Control lines were grown without FLC (C1, C2, and C3); lines grown in the presence of FLC at a concentration of 8 μg/mL (F1, F2, and F3). Step 2, confirmation of the FLC resistance stability through passages on a YPD plate in the absence of the drug and testing of single random colonies of each evolved replicate. Step 3, evaluation and validation of the selected single colony from each evolved replicate displaying maximum MIC_50_. (B) Drug susceptibility of the FLC-evolved replicates was tested by the microdilution assay. (C) Drug susceptibility determination by spot assays on solid agar YPD plate. The optical density of overnight grown cells at 600 nm was adjusted to 0.1 and serially diluted 5-fold up to five dilutions, which were spotted onto YPD plates with or without the drug. Growth differences were recorded following incubation of the plates for 48 h at 30°C.

A drug-susceptible C. auris isolate was exposed to MIC_50_-level concentrations of FLC through serial passages for 100 generations for 30 days, resulting in adapted cells showing high resistance to the drug. For the experimental evolution, a single colony of the parental progenitor isolate of C. auris (CBS10913T) belonging to clade 2 was used (Fig. 1A; also see Materials and Methods). The drug-susceptible cells were continuously exposed to 8 μg/mL of FLC, corresponding to its MIC_50_. The drug-containing replicates were referred to as F1, F2, and F3, while the replicates without the drug that served as controls were designated C1, C2, and C3. After 100 generations of growth in the presence or absence of FLC, the cells were assessed for their susceptibility to azoles. The replicates F1, F2, and F3, compared to C1, C2, and C3, acquired resistance to a high concentration of FLC, and their MIC_50_ values were 128, 64, and 256 μg/mL, respectively. To test if the replicates stably evolved to higher MICs, each FLC-resistant (FLC^r^) replicate was passaged daily on yeast extract-peptone-dextrose (YPD) plates without the drug for 30 days along with the fluconazole-sensitive control (C^s^) replicates grown in parallel. The FLC^r^ replicates, after 30 passages, were found to stably maintain their high MIC_50_ against FLC compared to the control susceptible replicates. Seven single random colonies from each stably adapted FLC^r^ replicates after 30 passages were picked up for analysis and tested for FLC susceptibility. Notably, single colonies from each passaged replicate continued to show high MICs, albeit to various thresholds, implying heterogeneity at the level of acquired resistance among adapted colonies. For instance, MIC_50_ values of the F1 replicate’s single colonies ranged between 64 and 128 μg/mL, while for F3 colonies, these values ranged between 128 and 256 μg/mL. In contrast, F2 colonies did not display much variation in MICs, and all seven colonies showed a consistent MIC_50_ of 64 μg/mL (see Table S2 in the supplemental material).

10.1128/mbio.03052-22.5 TABLE S2 MICs of different colonies of the evolved strains. Download Table S2, DOCX file, 0.01 MB. Copyright © 2022 Narayanan et al. 2022 Narayanan et al. https://creativecommons.org/licenses/by/4.0/ This content is distributed under the terms of the Creative Commons Attribution 4.0 International license.

For further analysis, we selected one colony displaying the highest MIC_50_ from each replicate (F1.2, F2.6, and F3.1). These selected azole-resistant colonies, F1.2, F2.6, and F3.1, stably exhibited higher MIC_50_s of 16-fold, 8-fold, and 32-fold toward FLC, respectively, with respect to the parent strain (Fig. 1B). The heterogeneity in the evolved isolates was also evident from the growth assay and the spot dilution test, as the three strains displayed different growth patterns in the presence of FLC, while they exhibited similar growth patterns in the absence of the drug (Fig. 1C and D). FLC-evolved C. auris replicates were also analyzed for their drug susceptibility toward other antifungal classes. While all the FLC^r^ replicates turned out to be resistant to other tested azoles such as ketoconazole, voriconazole, miconazole, and itraconazole (Fig. S1A), no collateral cross-resistance to other classes of drugs like polyene or echinocandins was exhibited by these FLC^r^-resistant colonies, implying antifungal class-specific attributes (Fig. S1B).

10.1128/mbio.03052-22.1 FIG S1 Evolved strains show resistance to azoles other than FLC but not polyenes or echinocandins. (A) Growth kinetic assays with and without the drug were performed using a microcultivation method in a 96-well plate in YPD medium at 30°C. Line plots depicting the growth of the evolved isolates F1.2, F2.6, and F3.1, along with the control strain C1.1, in the presence of. (B) Different azoles, namely, ketoconazole (KTZ) (B, panel a), miconazole (MCZ) (B, panel b), itraconazole (ITZ) (B, panel c), and voriconazole (VRZ) (B, panel d) are shown. (C) Growth of the evolved strains in the presence of a polyene, amphotericin B (AmB) (C, panel a), and an echinocandin, caspofungin (CSF) (C, panel b). The drug concentrations are shown on the *x* axis, and the growth is shown on the *y* axis as a relative (percent) change in growth. Download FIG S1, TIF file, 0.7 MB. Copyright © 2022 Narayanan et al. 2022 Narayanan et al. https://creativecommons.org/licenses/by/4.0/ This content is distributed under the terms of the Creative Commons Attribution 4.0 International license.

### Changes in global gene expression patterns in evolved fluconazole-resistant isolates reveal multiple ways of acquiring antifungal drug resistance by C. auris.

We performed a global transcriptomic analysis in FLC-evolved replicates to explore potential mechanisms by which the adapted cells acquire sustained increased resistance toward azole drugs. Three independent single colonies from each adapted replicate (F1.2, F2.6, and F3.1) were subjected to RNA sequencing. The total RNA was extracted from all three adaptors and control cell populations growing exponentially in the YPD medium in the absence of FLC to ensure that the transcriptome changes observed originated from stable transcriptional alterations. Each replicate was compared with the azole-susceptible C. auris control, which was parallelly run for 100 generations in the absence of the drug.

**FIG 2:**
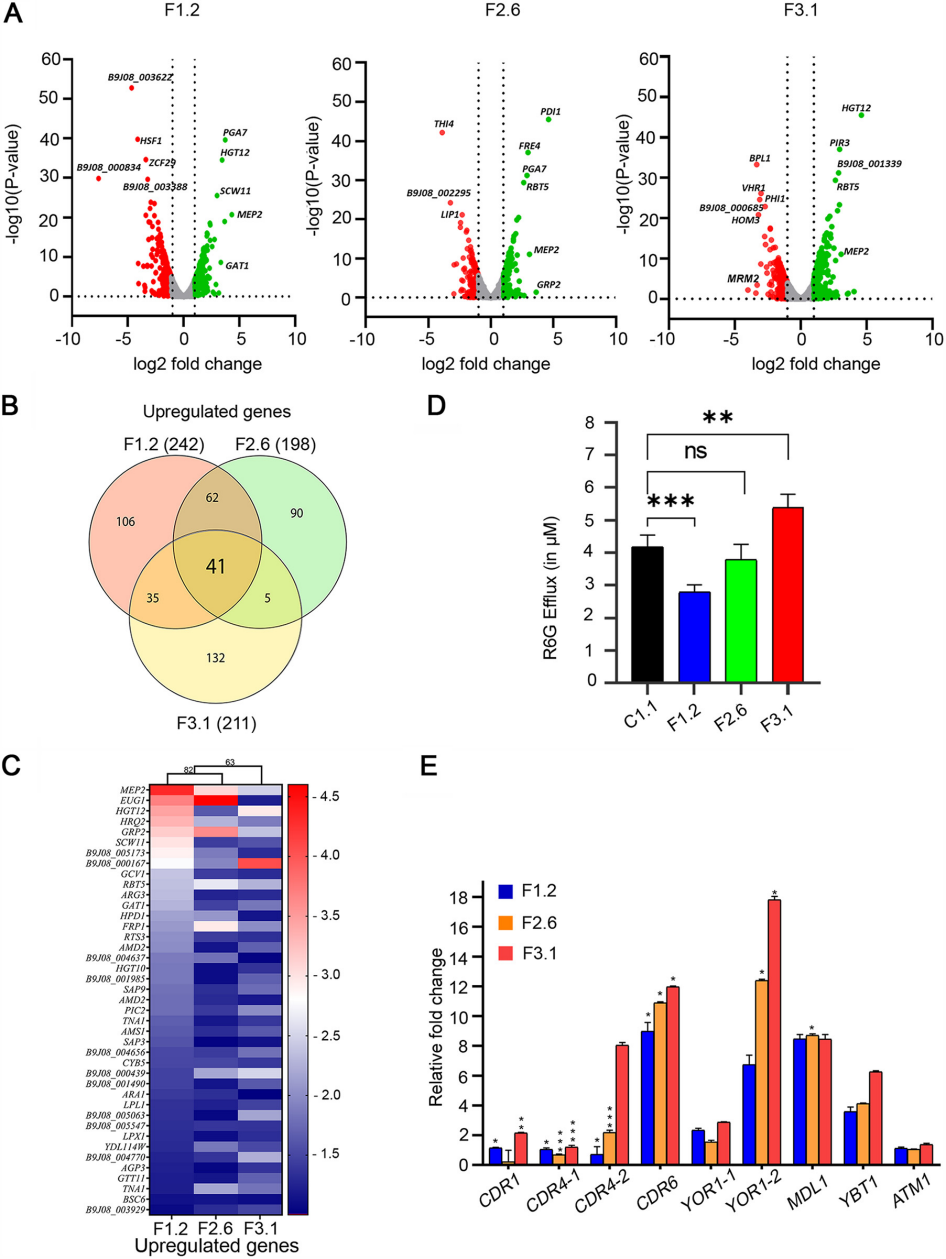
Transcriptomic analysis revealed the upregulation of drug efflux pumps in F3.1. (A) FLC-evolved replicates show differentially expressed genes; upregulated (green), downregulated (red), and neutral (gray) genes are marked. A few of the major altered genes are shown. (B) Venn diagrams showing the common upregulated genes in F1.2, F2.6, and F3.1 replicates. (C) Heatmap of the common upregulated genes representing the expression of each gene in all evolved replicates (as log_2_ fold change). (D) Bar graph representing the levels of efflux of fluorescent rhodamine 6G after 45 min of incubation with FLC in evolved and control cells. (E) Validation of transcriptional upregulation of selected ABC transporter genes in FLC-evolved replicates using real-time quantitative PCR. The experiments were performed in biological duplicates and technical triplicates. Level of significance is shown by the asterisks. ns, nonsignificant.

We considered a 2-fold threshold for differentially expressed genes with an associated *P*-value of ≤0.05 as significant for our analysis. The global transcriptional landscape of all three evolved replicates displayed varied responses in terms of the total number of genes with altered expression and their associated function (Fig. 2A and B; Data Set S1). The 41 commonly upregulated genes in all the three adapted FLC^r^ replicates included an ammonium transporter (*MEP2*) (19-fold change in the adapted cells) and a glucose importer (*HGT12*) (10-fold change in the adapted cells), along with an oxidoreductase (*GRP2*), secreted aspartyl proteases (*SAP9*, *SAP3*) and cell wall-related genes like *RBT5* and *PGA7* (Fig. 2C). Analyzing the pathways associated with the common differentially regulated genes revealed that most of the annotated upregulated genes belong to the class transporters. Ribosome biogenesis and RNA metabolism-associated genes constituted the majority of the 72 commonly downregulated annotated genes (Fig. S2A). Our transcriptomic data also show that several sugar transporter genes (HGT) are among highly overexpressed genes in all the adaptor replicates. Understanding how sugar importers contribute to azole resistance requires further investigation.

10.1128/mbio.03052-22.2 FIG S2 Possible mechanisms operating in acquired FLC resistance. (A) Venn diagram depicting the number of downregulated genes in the three evolved strains. (B) Heatmap showing the gene expression patterns of the common downregulated genes. (C) GO analysis of the common upregulated and downregulated genes. (D) Spot assay on the solid agar medium, showing increased susceptibility of F1.2 and F2.6 replicates towards myriocin (MYR) (inhibitor of the sphingolipid biosynthesis), selective susceptibility of F2.6 towards FK506 (calcineurin inhibitor), and the general resistance to fluconazole (FLC). Download FIG S2, TIF file, 1.1 MB. Copyright © 2022 Narayanan et al. 2022 Narayanan et al. https://creativecommons.org/licenses/by/4.0/ This content is distributed under the terms of the Creative Commons Attribution 4.0 International license.

Since efflux pumps are prominent drivers of fungal azole resistance and a strong correlation between the ABC transporter overexpression and azole resistance has been noted previously in C. auris (19), we performed a rhodamine 6G efflux assay to understand the role of ABC transporters in the acquired FLC resistance. We observed that the F3.1 replicate displayed higher extracellular levels of R6G than the control replicate. In contrast, low levels of extruded extracellular R6G were recorded for F1.2, while F2.6 did not show any significant change compared to the control replicate (Fig. 2D). This observation agreed with the maximum fold changes in the transcript levels observed in the RNA-seq data of the major drug exporter genes in F3.1 cells. Noticeably, 34 putative transporter genes were among the 211 upregulated genes, where ABC transporters genes like *CDR1*, *CDR4*, and *ATM1* and MFS transporters like *FLU1*, *HGT12*, *QDR1*, *MAL31*, *TNA1*, *HXT5*, *FLR1*, *HXT10*, *TPO4*, and *TNA1* were selectively upregulated in F3.1. The upregulation of some of these transporters (*CDR1*, *CDR4-1*, *CDR4-2*, *CDR6*, *YOR1-1*, *YOR1-2*, *MLD1*, *YBT1*, and *ATM1*) was validated by reverse transcriptase quantitative PCR (RT-qPCR), as depicted in Fig. 2E.

Previous studies suggest the involvement of calcineurin and sphingolipid pathways in increased drug resistance in C. albicans and other fungi (20, 21). F1.2 and F2.6 exhibited sensitivity to myriocin, a potent inhibitor of sphingolipid biosynthesis (Fig. S2B). In addition, F1.2 showed a significantly reduced expression of the transcription factor gene *HSF1*, which regulates *HSP90* and calcineurin signaling cascades governing azole resistance (22). F2.6 was also found to be susceptible to the immunosuppressant FK506, a calcineurin inhibitor (Fig. S2B). Taken together, the downregulation of the *HSF* and *HSP* genes, as well as the increased susceptibility toward FK506 and to myriocin in the evolved strains, we propose that both pathways may be involved in regulating azole resistance of C. auris. While the role of sphingolipids and calcineurin cascades in influencing drug resistance is reported in fungi (23), their possible rewiring and impact on C. auris remain to be validated. The individual replicate, F3.1, also exhibited differential expression of separate sets of genes involved in metabolic processes like cellular stress, transport, lipid metabolism, etc. In summary, the gene expression analysis suggested multiple pathways operating in acquiring azole resistance in the evolved isolates.

### Whole-genome sequencing hints at aneuploidy in the evolved strains.

To understand the changes that occurred at the genomic DNA sequence level, whole genomes of the F series were sequenced along with the control strain. The Illumina sequencing raw reads were then aligned to clade 1 reference assembly of C. auris (http://www.candidagenome.org/) for variant calling. From the single nucleotide polymorphism (SNP) data, nonsynonymous changes that occurred in the exons in the evolved isolates were extracted and compared with the control strain to subtract the background SNPs (see Materials and Methods). The common SNPs identified in all three evolved strains and strain-specific nonsynonymous SNPs are listed in Data Set S2. We detected a nonsynonymous mutation in *TAC1* (V188A) in F3.1, but not in F1.2 and F2.6 replicates. Finally, F3.1 also showed an increased expression of *CDR1* followed by enhanced substrate efflux, while none of the other two replicates showed such changes. However, no mutation in *ERG* genes was detected, and they are well-established drivers of FLC resistance.

**FIG 3:**
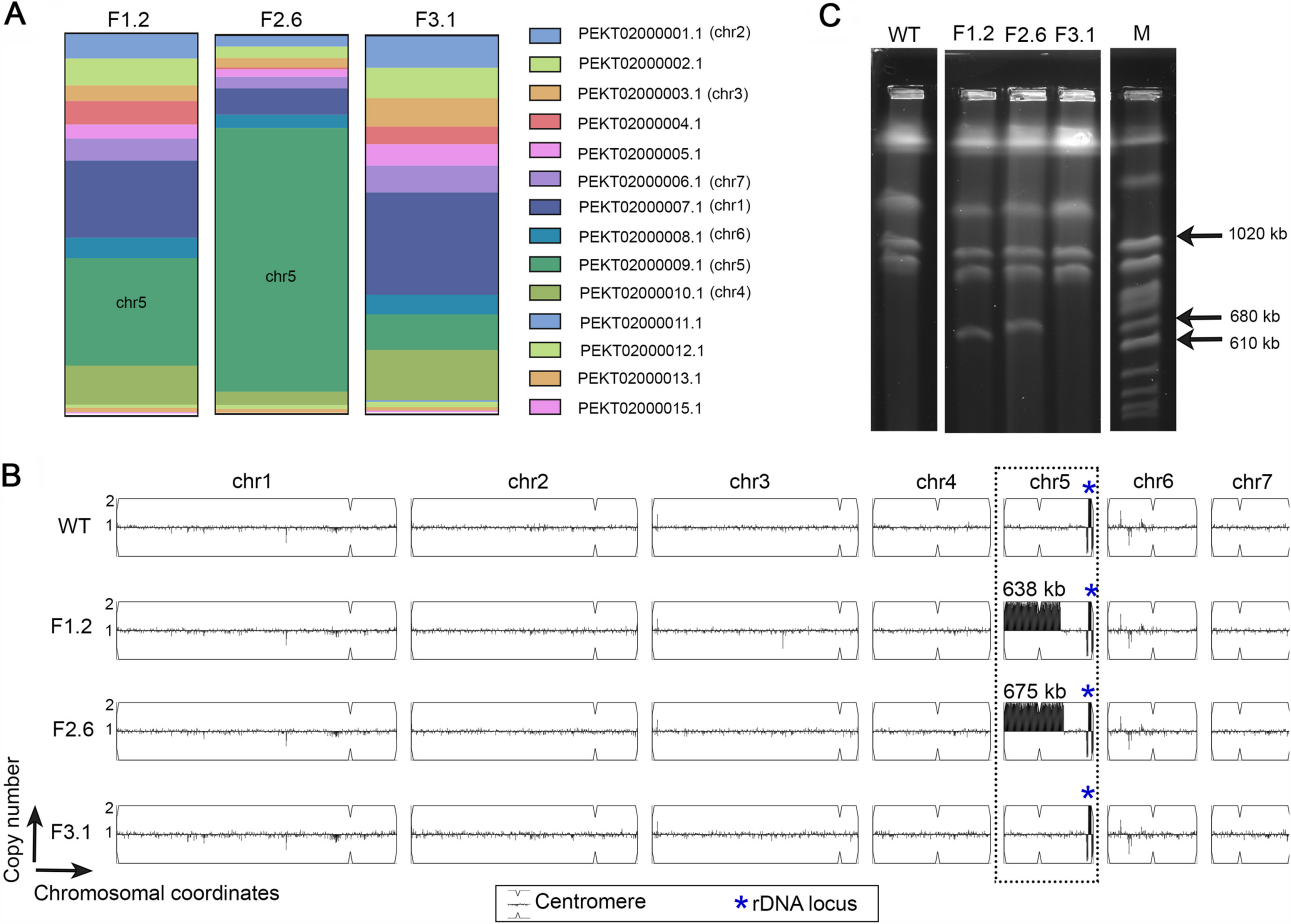
Directed evolution in the presence of FLC leads to the formation of a supernumerary chromosome in the C. auris clade 2 strain. (A) Plots depicting the percentage of upregulated genes in F1.2, F2.6, and F3.1 located in different scaffolds in the reference assembly accession no. GCA_002759435 (Candida Genome Database). (B) Segmental duplications detected by copy number variation analysis using YMAP. Chromosome-wise mapping of reads to the reference is shown for the parental strain and the three evolved azole-resistant strains. Chromosome positions are shown on the *x* axis, and the copy numbers are on the *y* axis. Chromosome 5, which underwent duplication, is indicated by the dotted box. Sizes of the duplicated regions are shown. Centromere positions on the chromosomes are marked. (C) Electrophoretic karyotyping of the parent strain (wild type [WT]) and the evolved azole-resistant strains (F1.2, F2.6, and F3.1) showing the additional chromosomes. S. cerevisiae chromosomes are used as size markers, shown as M. The size range of the supernumerary chromosomes is marked.

Ploidy changes have been implicated in azole resistance in many *Candida* spp. We analyzed the scaffold-wise distribution of upregulated genes in all three FLC-adapted isolates to detect if upregulated genes are clustered on any specific scaffold in each case. A higher proportion of upregulated genes in both F1.2 and F2.6 (28% and 70%, respectively) were located on the scaffold PEKT02000009 of the genome assembly (Candida Genome Database), which corresponds to chr5 in C. auris. Most upregulated genes in F3.1 were present on the scaffold PEKT02000007, corresponding to chr1 of the same organism (27%) (Fig. 3A).

The raw reads of whole-genome sequencing were also mapped to the clade 2 reference genome (GenBank assembly accession no. GCA_003013715) using YMAP (http://lovelace.cs.umn.edu/Ymap/) that detected two segmental duplications as distinct peaks, one each in F1.2 and F2.6. Both duplications occurred in chr5, including the centromere sequence (24) (Fig. 3B), resulting in the duplication of several genes. An additional peak was also present in the wild type and marked the location of rRNA genes in the C. auris genome. The duplication in chr5 in F1.2 spanned a length of 639 kb (1 to 638,574 bp), while duplication in F2.6 spanned 675 kb (1 to 674,550 bp).

### Evolved azole resistance strains harbor supernumerary centric chromosomes.

Analysis of the whole-genome sequencing data revealed the possible occurrence of segmental aneuploidy through chromosomal duplication that included the centromere sequence of chr5. If the duplicated centromere sequences happen to be present on the same chromosome, the resulting dicentric chromosome is likely to be unstable. Alternatively, the duplicated sequence may exist as a supernumerary chromosome of reduced length. To verify if such a duplication with a centromere sequence can exist as a separate chromosome, we performed electrophoretic karyotyping of the three evolved strains exhibiting azole resistance. Strikingly, this analysis revealed the presence of an additional chromosome in each of the two isolates, F1.2 and F2.6 (Fig. 3C). The length of the duplicated sequences identified by the CNV analysis corresponded to the sizes of the additional chromosomal bands in the pulsed-field gels, confirming that they exist as linear centric chromosomes. No additional chromosomes were observed in the wild-type control or F3.1. Combined with the sequencing results, it was evident that the centromere-inclusive segmental duplications from chr5 detected in the copy number analysis exist as supernumerary chromosomes (SNCs) in the strains, in addition to the seven core chromosomes. Multiple colonies of both F1.2 and F2.6 were tested to confirm the presence of the additional chromosomes.

### Supernumerary chromosomes are directly linked to azole resistance.

**FIG 4:**
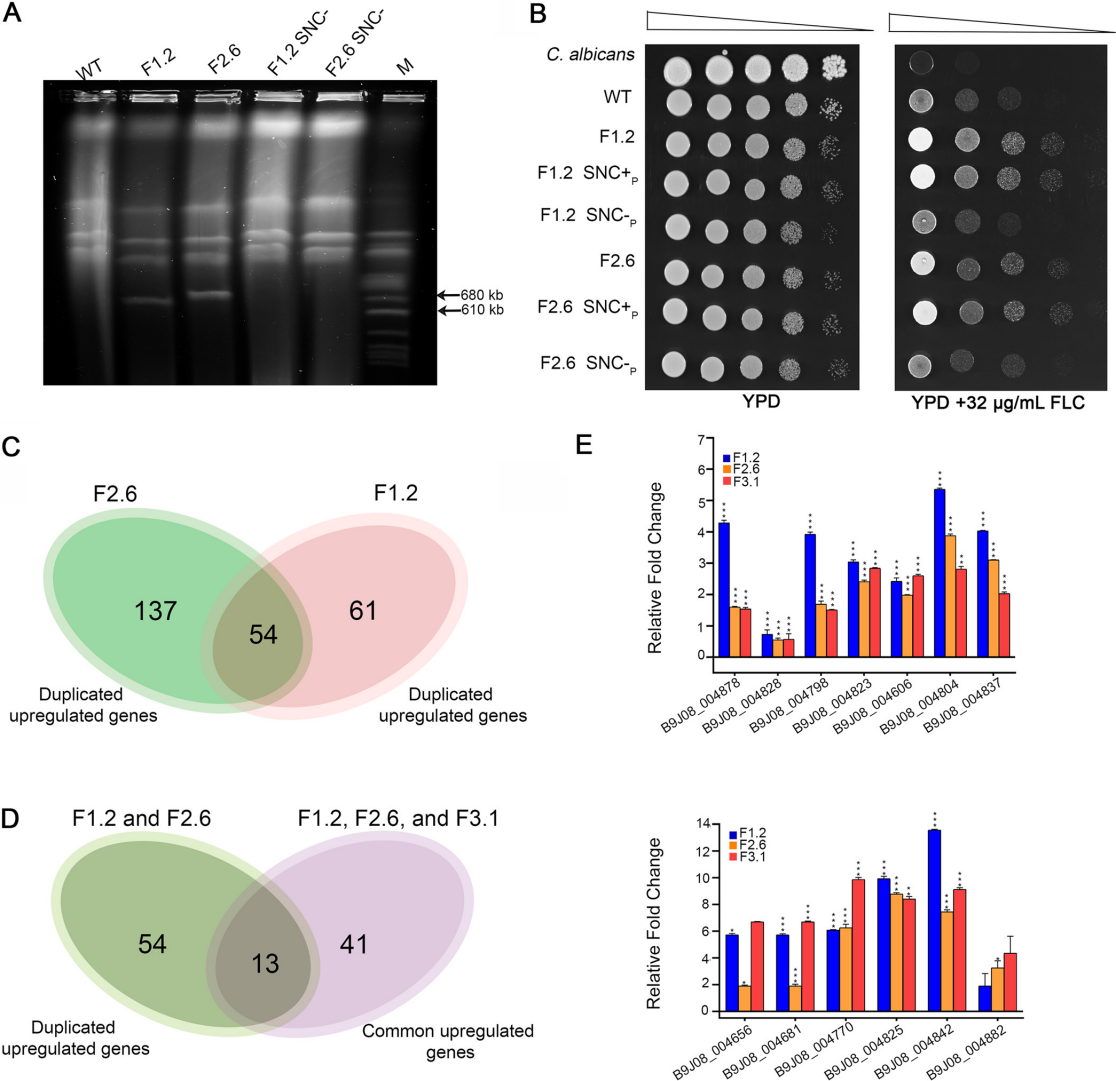
Potential drug resistance genes in C. auris. (A) Serial passage of the evolved strains, F1.2 and F2.6, leads to supernumerary chromosome loss in two colonies, shown as F1.2 SNC- and F2.6 SNC-_p_. (B) Dilution spot assay showing the effect of supernumerary chromosome on FLC resistance. (C) Comparison of duplicated and upregulated genes in F1.2 and F2.6 identifies an overlap of 54 genes. (D) Overlap of 13 genes obtained by comparing 54 genes in panel C with the upregulated genes in all the evolved strains. (E) Bar graphs depicting gene expression patterns of the 13 shortlisted genes in the 3 evolved isolates. The experiments were performed in biological duplicates and technical triplicates. C. auris gene names are marked on the *x* axis, and the relative fold change is on the *y* axis.

As SNCs appeared during the experimental evolution in the presence of FLC, we sought to understand if they confer resistance to azoles by the duplication of azole resistance-associated genes. To investigate the role of the SNCs, we passaged F1.2 and F2.6, the FLC-adapted isolates from the experimental evolution experiment in YPD without FLC for 100 generations at two different temperatures, 30°C and 37°C. Karyotypes of five different colonies from all the terminal lines grown at both temperatures were analyzed. No loss of SNCs was observed at 30°C in this short experimental evolution regime. However, one colony out of five tested lost the SNC at 37°C in both the strains, F1.2 and F2.6 (Fig. S3A; Fig. 4A). These two colonies (F1.2 SNC-_p_ and F2.6 SNC-_p_), when tested for FLC resistance, were found to have lost the acquired azole resistance and exhibited growth similar to that of the wild type on YPD plates supplemented with different concentrations of FLC (Fig. 4B), suggestive of the direct role of the SNCs in acquired FLC resistance. Two terminal colonies, one each from the F1 series and F2 series, that retained the chromosomes (F1.2 SNC^+^_p_ and F2.6 SNC^+^_p_) (Fig. S3A) were also tested, along with F1.2 SNC-_p_ and F2.6 SNC-_p_. These colonies retained the acquired FLC resistance, unlike F1.2 SNC-_p_ and F2.6 SNC-_p_ (Fig. 4B). This observation helped us rule out any possible effects of the serial passages in the absence of the drug on the reduction in the acquired drug resistance of the FLC-adapted strains observed after SNC loss. The strains harboring the SNC and the ones that underwent SNC loss were also tested in media supplemented with amphotericin B and caspofungin. No variations were observed in the tested strains (Fig. S3B), confirming that the effects of the chromosomes that originated during experimental evolution in the presence of FLC were restricted to that specific stress condition. The strains harboring additional chromosomes exhibited no visible growth defects in YPD at 30°C and 37°C.

10.1128/mbio.03052-22.3 FIG S3 The additional chromosome can be lost on serial passages in the absence of FLC. The evolved strains, F1.2 and F2.,6 were grown in YPD in the absence of FLC, and individual colonies from the terminal lines (S1 to S9 for F1.2 and S10 to S20 for F2.6) were screened for loss of the additional chromosome. The strains chosen for further experiments are labeled F1.2 SNC-_p_, F2.6 SNC-_p_, F1.2 SNC^+^_p_, and F2.6 SNC^+^_p_. Clade 2 parental strain (labeled P) was used as the control. S. cerevisiae chromosomes are used as size markers, shown as M. Size range of the supernumerary chromosomes is marked. (B) Dilution-spot assay showing the effect of SNC on amphotericin B (AmB) and caspofungin (CSF); the control plate (YPD) is shared with Fig. 4B. Download FIG S3, TIF file, 2.8 MB. Copyright © 2022 Narayanan et al. 2022 Narayanan et al. https://creativecommons.org/licenses/by/4.0/ This content is distributed under the terms of the Creative Commons Attribution 4.0 International license.

### A set of potential genes responsible for conferring azole resistance in C. auris.

The aneuploidy-dependent drug resistance mechanism relies primarily on the increased expression of genes due to their copy number variations (CNVs). On the other hand, the aneuploidy-independent mechanisms include overexpression of a set of genes by acquiring mutations. While the drug resistance could be acquired by related or unrelated pathways, we hypothesized that a common mechanism of drug resistance may exist irrespective of the presence of CNVs or mutations. To achieve this, we identified the list of common duplicated upregulated genes (DUGs) and common duplicated downregulated genes (DDGs) in F1.2 and F2.6 and found a set of 54 common genes as DUGs (Fig. 4C; Data set S3), while only one gene, B9J08_004664, a tRNA gene, constituted the DDGs. We also compared the list of 54 genes with the 41 genes that were upregulated across the 3 evolved strains. Thirteen genes, commonly upregulated in all the evolved lines compared to the control and duplicated in F1.2 and F2.6, were selected based on this analysis (Fig. 4D) and are listed in Table S4. RT-PCR confirmed the upregulation of the 13 genes in all three FLC-resistant isolates (Fig. 4E). However, further analysis is required to find whether other genes excluding the 13 that are listed are responsible for conferring drug resistance in a mode-specific (aneuploidy-dependent versus aneuploidy-independent) manner.

10.1128/mbio.03052-22.7 TABLE S4 Putative azole-resistance associated genes in C. auris. Download Table S4, DOCX file, 0.01 MB. Copyright © 2022 Narayanan et al. 2022 Narayanan et al. https://creativecommons.org/licenses/by/4.0/ This content is distributed under the terms of the Creative Commons Attribution 4.0 International license.

Of the 13 genes, the C. albicans homolog of B9J08_004681 and B9J08_004828, orf19.6502, and *GRP2*, respectively, were reported to be upregulated in FLC-resistant clinical isolates of C. albicans (25). Another major upregulated gene, *MEP2*, the ammonia transporter, is shown to be influenced by *GAT1*, a transcription factor, and positively regulates the filamentation growth of C. albicans cells under nitrogen-starved conditions via activation of the MAP kinase and cAMP-dependent signaling pathway (26). In Cryptococcus neoformans, lower nitrogen concentrations promote ergosterol and capsule biosynthesis (27). In C. albicans, the higher survival percentage in the presence of antifungal drugs under nitrogen-starved cultures suggests that nitrogen starvation influences the drug susceptibili/ty of cells (28). Our data showing overexpression of *MEP2* and its transcription factor *GAT1* among the highly overexpressed common genes in FLC-resistant isolates suggest its link to FLC resistance in C. auris.

## DISCUSSION

We chose a drug-susceptible C. auris clade 2 isolate as the starting strain to study the evolution of FLC resistance in the species. This strain was experimentally evolved at a constant drug pressure to yield three different drug-resistant colonies that exhibited heterogeneous FLC resistance. Both aneuploidy-dependent and aneuploidy-independent pathways were found to operate in the terminal lines, resulting in FLC resistance despite originating from the same parent strain propagated under similar culture conditions. Out of the three terminal lines, two underwent segmental duplications of the same chromosome, Chr5, that existed as separate centric chromosomes. The additional chromosomes were directly associated with azole resistance. The third line exhibited overexpression of drug efflux pumps, representing the aneuploidy-independent mode of acquiring FLC resistance. Based on the analyses, we shortlist a few genes and speculate that one or more genes, on overexpression, confer resistance to FLC. Both the mechanisms detected in the study were stable, as the evolved strains retained the acquired FLC resistance even after many passages in YPD, in the absence of drug stress.

Each of the three evolved lines had a unique MIC_50_ value, indicative of heterogeneity in the evolving population structure. Parallelly, FLC-adapted populations of C. albicans were also found to acquire different MICs, following distinct trajectories (5), emphasizing the importance of chance events that get selected under drug pressure. The observed heterogeneity possibly recapitulates the clinical scenario, as coisolates obtained from the same patient are known to behave differently. For instance, strains of Clavispora lusitaniae isolated from the right upper lobe and right lower lobe of the lung of a patient shared a common ancestor but exhibited different phenotypic and genomic DNA sequence features (29). Between the two C. auris isolates obtained from the right and left ear of a patient on the same date, 36 SNVs were detected despite belonging to the same lineage, and the two isolates exhibited different FLC resistance (30).

Chromosomal changes and resulting aneuploidy can be major drivers of antifungal drug resistance. Isochromosome formation leading to copy number variations in *TAC1* and *ERG11* leads to FLC resistance in C. albicans (31). Previous studies have indicated the role of chr5 aneuploidy in azole resistance in C. auris. Whole-chromosome duplication, detected as copy number variation, was reported in a clade 1 experimentally evolved isolate that conferred FLC resistance. It was also observed that on passaging in the absence of the drug, the CNV was lost, and the strain lost the acquired resistance (32). We observe that the duplication of chr5 is associated with FLC resistance in a clade 2 isolate in this study, suggesting that it is a conserved mechanism across clades involving a common set of genes harbored on chr5.

We show that a duplicated region of a genome may exist as a stable supernumerary chromosome if it carries a functional centromere sequence. This mitotic chromosome stability can also account for the FLC resistance that persisted after the passages of the evolved strains in the absence of the drug. Whole-chromosome duplication of chr5 is reported by independent studies in association with FLC resistance (11, 32, 33). This raises the possibility that the formation of the SNC proceeded through whole-chromosome duplication and subsequent sequence loss. Such centromere-inclusive segmental duplications existing as separate chromosomes conferring FLC resistance through copy number changes of specific genes are reported in C. glabrata (34). Duplicated genomic regions, if stabilized and fixed in the population, can act as sources of paralogs during evolution. They serve as the templates on which natural selection may act for functional innovation, which can be neofunctionalization, subfunctionalization, or gene dosage effects (35). It is also evident from the study that the segmental duplication of a chromosome, rather than the whole chromosome, is sufficient to impart azole resistance to the strain.

Out of the three evolved strains, F3.1 acquired fluconazole resistance through upregulation of ABC and MFS transporters. Out of them, *ATM1*, was recently revealed to confer ketoconazole resistance in Malassezia restricta (36). Among the MFS transporters, we also observed the upregulation of a few that belong to the drug/H^+^ antiporter (DHA) group with established roles as multidrug/multixenobiotic efflux pumps. For instance, while *FLU1* has been shown to be marginally influencing azole resistance (37), *FLR* genes are also known azole resistance contributors (38). CgQdr2 has also been demonstrated to confer azole resistance (39). Taken together, the F3.1 cells seem to rely more on drug export in evolving to FLC resistance than the parallelly run F1.2 and F2.6 replicates, which exhibit aneuploidy-dependent FLC resistance. This also indicates that independent colonies traversed through distinct trajectories though grown under the same conditions to attain different levels of FLC resistance. Most noteworthy among other upregulated transporters are sugar transporters, whose role as drug importers has been explored recently (40). The role of highly expressed sugar importers that might improve fitness and contribute to drug resistance requires further study.

Whole-chromosome duplication and segmental aneuploidy of chr5 previously reported in C. auris (33) conferred FLC resistance, which was attributed to copy number variations of genes, including *TAC1B*, the zinc-cluster transcription factor that positively regulates drug efflux pumps such as *CDR1*. Though we observed duplication of *TAC1B* in F1.2 and F2.6, as it is located on the SNC, we did not observe any upregulation of the gene or drug pumps like *CDR1* in RNA-seq data. However, a nonsynonymous mutation (V188A) in *TAC1* was identified in the evolved strain F3.1. *CDR1* was upregulated only in this strain, suggesting that the amino acid substitution might have a possible role in acquired azole resistance. *In vitro* evolution of a drug-susceptible strain of C. auris in the presence of fluconazole was previously shown to select for missense mutations (R495G and F241S) (12) and codon deletions (ttcagt/agt) (11) in *TAC1B*. FLC-resistant C. auris clinical isolates were also found to harbor missense mutations in *TAC1* (S611P) (41). Whole-genome sequencing of the three evolved strains revealed the presence of common SNPs and strain-specific SNPs. Analysis of amino acid sequences across different assemblies available in the public domain suggests that other amino acid changes identified as common SNPs are probably polymorphic sites and are likely not directly involved in conferring azole resistance.

**FIG 5:**
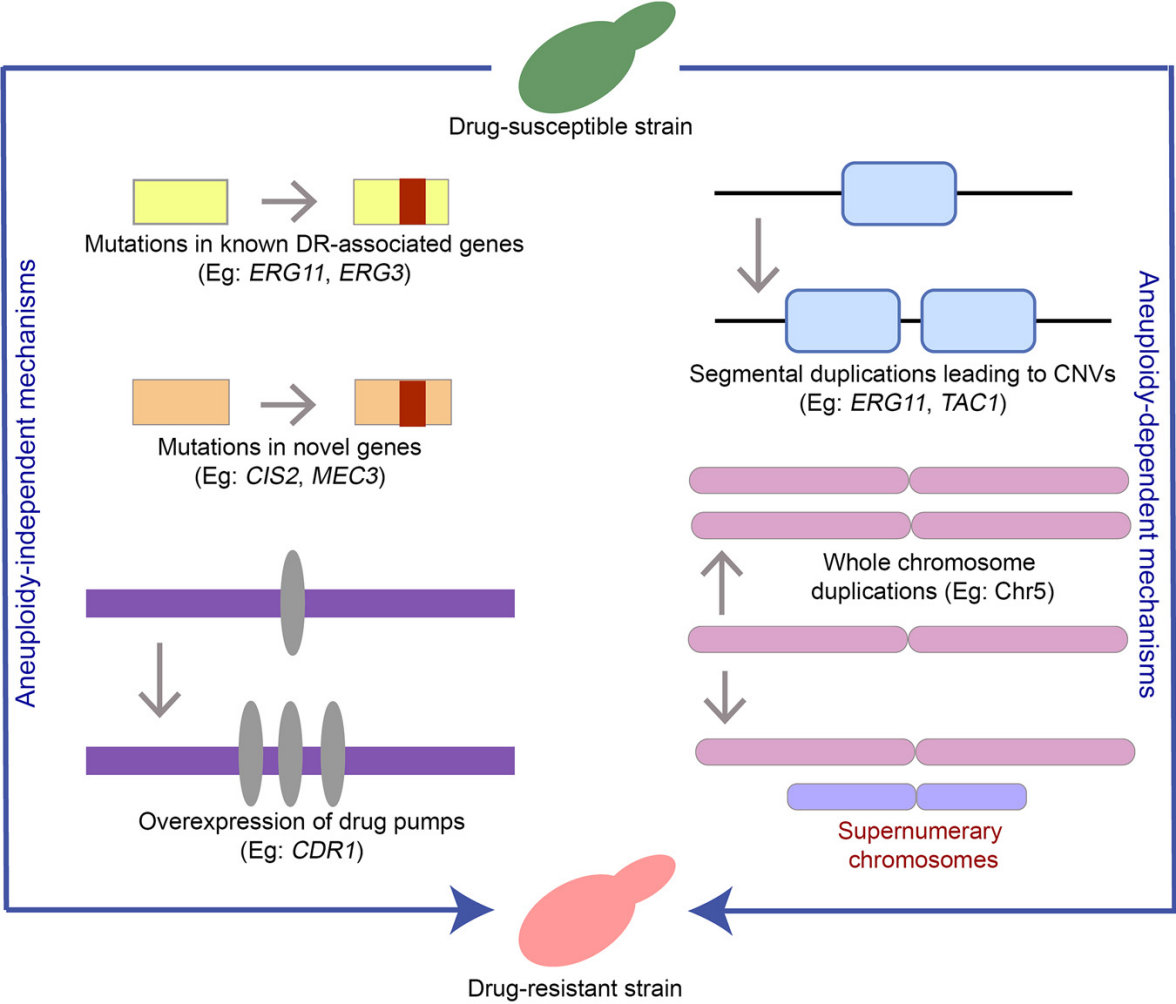
Schematic summarizing different pathways leading to FLC resistance in C. auris. Aneuploidy-independent mechanisms involve mutations in known drug resistance (DR)-associated genes (8, 11, 19, 41), mutations in novel genes (11), and overexpression of drug efflux pumps (19, 55). Copy number variations (CNVs) can result from segmental duplications (11), whole-chromosome duplications (32, 33), or additional chromosomes (this study). These collectively represent the aneuploidy-dependent mechanisms that render a strain FLC resistant.

In summary, our study adds to the existing knowledge of multiple pathways leading to FLC resistance in C. auris (Fig. 5), which includes aneuploidy-independent and aneuploidy-driven mechanisms. Further characterization of selected genes that were similarly altered in the evolved lines is required to understand their role in azole resistance. Our study focused on the changes in a drug-susceptible strain under constant azole stress in a rich medium. Identification of events like supernumerary chromosome formation in clinical isolates will provide us with more information on the frequency at which they occur in natural populations in host niches. Studying these events in drug-susceptible isolates across clades will also help establish the common trajectories culminating in azole resistance.

## MATERIALS AND METHODS

### Strains and media.

We procured C. auris clade 2 isolate (CBS10913T) from the Central Bureau voor Schimmel Cultures (CBS), Fungal Biodiversity Centre of the Royal Netherlands Academy of Arts and Sciences (KNAW), Utrecht. At every transfer, the ancestor and the evolved strains were stored at −80°C in 50% glycerol and grown in YPD (1% yeast extract, 2% peptone, and 2% dextrose) at 30°C when revived. Different antifungal drugs in various concentrations were added to YPD agar (as mentioned in the corresponding figure legends) for spotting assays. C. albicans strain SC5314 was also used as a control in this study. All the strains used in this study are listed in Table S1 in the supplemental material.

10.1128/mbio.03052-22.4 TABLE S1 Strains used in the study. Download Table S1, DOCX file, 0.01 MB. Copyright © 2022 Narayanan et al. 2022 Narayanan et al. https://creativecommons.org/licenses/by/4.0/ This content is distributed under the terms of the Creative Commons Attribution 4.0 International license.

### Experimental evolution of C. auris CBS10913T.

For experimental evolution, C. auris strain CBS10913T (MIC_50_s, FLC, 8 μg/mL; AmB, 0.5 μg/mL; and caspofungin [CSF], 0.1 μg/mL) was used. The protocol of experimental evolution described earlier (28) was adapted with some modifications. The strain was revived from a frozen stock on a YPD plate and incubated for 48 h at 30°C. Thereafter, a single colony was patched on a new YPD plate and incubated for another 48 h at 30°C. A single colony from this plate was cultured in a fresh 10 mL YPD broth and incubated for 72 h at 30°C. From the stationary culture, 10 μL of cells with an optical density (OD) of 0.1 (OD_600_) cells were transferred into three independent tubes, each containing 9,990 μL fresh YPD with or without FLC (8 μg/mL). Additionally, one tube was kept as a medium control. Initially, all the replicates and controls were incubated for 72 h at 30°C (Fig. 1A). The culture from each replicate and control was transferred into fresh YPD broth (with or without the drug, 10 μL from the previous culture in 9,990 μL fresh YPD broth, resulting in 1:1,000 dilution) and incubated for another 72 h at 30°C. After 72 h (10 generations), adapted cells with positive control were transferred into fresh media again in a 1:1,000 dilution. One such transfer of 1:1,000 dilution at 30°C for 72 h corresponds to 10 generations [log_2_(1,000) = 9.97; 1 transfer is equivalent to approximately 10 generations]. For 100 generations, 10 such transfers of the cells were performed in the presence or absence of FLC. After every 10 generations, an aliquot of cells was drawn and stored at −80°C for further analysis.

For the supernumerary chromosome loss assay, the evolved strains harboring the additional chromosome were passaged in YPD without fluconazole. We used 10 μL of an overnight culture derived from a single colony to inoculate two sets of 9,990 μL of fresh YPD; one was grown at 30°C and the other at 37°C for 24 h to saturation. Ten such passages were done for both strains F1.2 and F2.6. The terminal lines (after passage 10) were streaked on YPD plates to obtain single colonies, which were then subjected to electrophoretic karyotyping.

### Growth assays.

The growth kinetics assays were performed by a microcultivation method in 96-well plates using liquid handling system (Tecan, Austria) in YPD broth at 30°C. Briefly, overnight-grown yeast cultures were diluted to an OD of 1.0 (*A*_600_), and 20 μL of each culture was mixed with 180 μL YPD broth with the selected antifungals in a 96-well plate. The OD was measured at 600 nm every 30 min for 48 h.

### Determination of MICs and spot assays.

C. auris cells were grown overnight at 30°C in YPD media, and MIC assays were performed as per Clinical and Laboratory Standards Institute (CLSI) guidelines (42). The cells were diluted in 0.9% saline solution to obtain an OD (*A*_600_) of 0.1. The cells were then diluted 100-fold in the YPD medium. The diluted cell suspension was added to the wells of round-bottomed 96-well microtiter plates containing equal volumes of media and different concentrations of the drug. The plates were incubated at 30°C for 48 h. The MIC test endpoint was evaluated by measuring the optical density at 600 nm in a microplate reader (Bio-Rad iMark) and was defined as the lowest drug concentration that gave 50% inhibition of growth (MIC_50_) compared to the growth of the drug-free control.

### R6G efflux assay.

The rhodamine 6G (R6G) efflux assay was performed by the energy-dependent efflux method. In this assay, C. auris cells from an overnight culture were inoculated in fresh YPD medium at an OD of 0.1 and grown for 4 to 5 h at 30°C until the log phase. The cell suspension was washed with 1× phosphate-buffered saline (PBS) twice and incubated for 3 h at 200 rpm and 30°C for starvation (glucose free) to reduce the activity of the ABC transporters. After incubation, cells were washed twice with PBS and diluted to obtain 10^8^ cells/mL in PBS. R6G, at a final concentration of 10 μM, was added to the suspension and incubated for 3 h at 30°C and 200 rpm for the accumulation assay. For the efflux assay, the cells were washed twice in PBS, 2% glucose was added to the suspension, and the suspension was incubated for 45 min. The supernatant was then collected, followed by measurement of fluorescence of R6G in a fluorescence spectrophotometer at excitation and emission wavelengths of 527 nm and 555 nm, respectively.

### RNA isolation.

A saturated overnight culture was used to inoculate 10 mL of fresh YPD at an OD of 0.1, which was grown for 4 to 5 h at 30°C to obtain a log-phase culture. The cells were then collected by centrifugation and washed with diethyl pyrocarbonate (DEPC)-treated water. Total RNA was isolated using RNeasy minikit (Qiagen, Hilden, Germany; catalog no. 74104), following the manufacturer’s specifications. Total RNA in the samples was quantified using NanoDrop 2000 spectrophotometer (Thermo Scientific, USA).

### RNA sequencing and analysis.

RNA sequencing libraries from the extracted RNA were prepared with NEBNext Ultra II directional RNA library prep kit (New England Biolabs, MA, USA) at Genotypic Technology Pvt. Ltd., Bangalore, India. mRNA isolation, fragmentation, and priming were performed using 500 ng of the total RNA as the starting material. Fragmented and primed mRNA was further subjected to first-strand synthesis followed by second-strand synthesis. The double-stranded cDNA was purified using JetSeq magnetic beads (Meridian Bioscience; catalog no. BIO-68031). Purified cDNA was end repaired, adenylated, and ligated to Illumina adapters as per NEBNext Ultra II directional RNA library prep protocol, followed by second-strand excision using USER enzyme at 37°C for 15 min. Adapter-ligated cDNA was purified using JetSeq beads and was subjected to 11 cycles for indexing (98°C for 30 s, cycling of 98°C for 10 s and 65°C for 75 s, and 65°C for 5 min) and enriching the adapter-ligated fragments. Final PCR products (sequencing libraries) were purified with JetSeq beads, followed by a library quality control check. Illumina-compatible sequencing libraries were quantified by Qubit fluorometer (Thermo Fisher Scientific, MA, USA), and fragment size distribution was analyzed on an Agilent 2200 tape station. The libraries were paired-end sequenced on an Illumina HiSeq X Ten sequencer (Illumina, San Diego, USA) for 150 cycles following the manufacturer’s instructions.

FastQC (https://www.bioinformatics.babraham.ac.uk/projects/fastqc/) was used to process the raw reads for quality assessment and preprocessing, which includes removing the adapter sequences and low-quality bases (<q30) using TrimGalore3 (https://www.bioinformatics.babraham.ac.uk/projects/trim_galore/). The preprocessed high-quality data were aligned to the reference genome (Candida Genome Database; Candida auris strain B8441) using Bowtie24 (43) with the default parameters to identify the alignment percentage. Reads were classified into aligned reads (which align to the reference genome) and unaligned reads. HTSeq5 (44) was used to estimate and calculate gene abundance. Absolute read counts for genes were identified and used in differential expression calculations. DESeq6 (45) was used to identify the differentially expressed genes. Genes were categorized into up, down, and neutrally regulated based on the log_2_ fold change cutoff of 1. DESeq-normalized expression values were used to calculate fold change for a given gene. The regulation for each gene was assigned based on log_2_ fold change. The genes which show log_2_ fold change less than −1 are represented as downregulated, the values greater than 1 are represented as upregulated, and between −1 to 1 are termed as neutrally regulated.

### Gene Ontology (GO) and pathway analysis.

Genes were annotated for functional information using BLAST7 (46) homology search against Candida albicans protein sequences from the UniProt database. Genes were assigned with a homolog protein from the reference organism if the match was found at an E value less than e-5 and minimum similarity greater than 30%. Pathway analysis was done using the KAAS8 server (47). Pathways from reference organisms were considered a reference for pathway identification. Compiled pathways per gene were mapped to the differentially expressed genes.

### Quantitative real-time PCR and analysis.

Total RNA was isolated as described above and was quantified using NanoDrop 2000 spectrophotometer. Approximately1 μg RNA was taken for cDNA synthesis performed using the RevertAid H Minus first strand cDNA synthesis kit (Thermo Fisher Scientific, Waltham, MA, USA; catalog no. K2562) according to the manufacturer’s instructions. iTaq Universal SYBR green supermix (Bio-Rad; catalog no. 172-5124) was used, along with the desired gene-specific oligonucleotide primers (Table S3), to evaluate the quantitative expression profile after normalization with the housekeeping gene CauTDH3 using CFX96 real-time PCR system (Bio-Rad, USA). The gene expression level was measured by calculating the threshold cycle (*C_T_*) value of the *CauTDH3* gene and the desired genes. Comparative gene expression profiles were measured by the 2^−ΔΔ^*^CT^* method. qRT-PCR was performed in biological duplicates and technical triplicates.

10.1128/mbio.03052-22.6 TABLE S3 List of primers used in this study. Download Table S3, DOCX file, 0.01 MB. Copyright © 2022 Narayanan et al. 2022 Narayanan et al. https://creativecommons.org/licenses/by/4.0/ This content is distributed under the terms of the Creative Commons Attribution 4.0 International license.

### Genomic DNA isolation.

Genomic DNA was extracted from the cells grown in YPD liquid using the Qiagen yeast DNA kit (QIAamp DNA minikit; catalog no. 51304) according to the manufacturer’s instructions. Genomic DNA was then eluted with distilled water, and concentration (absorbance at 260 nm) and purity (absorbance at 260 nm/280 nm) were checked using NanoDrop 2000 spectrophotometer. Whole-genome sequencing was performed at Clevergene Biocorp Pvt Ltd., Bangalore, India.

### Whole-genome sequencing and data analysis.

For genome sequencing, a paired-end library with an average insert size of 300 bp was prepared and sequenced using the Illumina NovaSeq 6000 platform. Data quality was checked using FastQC and MultiQC (48). The data were checked for base call quality distribution, percentage of bases above Q20, Q30, GC percent, and sequencing adapter contamination. All the samples passed the quality control (QC) threshold (Q20 > 95%). Raw sequence reads were processed to remove adapter sequences and low-quality bases using fastp (49).

### Alignment, variant calling, and variant annotation.

The trimmed reads were aligned to the reference genome of Candida auris strain B8441 (http://www.candidagenome.org/download/sequence/C_auris_B8441) using the BWA-MEM algorithm (50). The alignments were processed to remove PCR duplicates using SAMtools (51). The base qualities were recalibrated, and variants were called using freebayes (https://github.com/freebayes/freebayes) with haplotype 1 and a quality score of more than 20. The variants were annotated using snpEff (52) using the annotation of their respective reference genomes. The VCF files were processed using snpSift (53) to convert the data into tab-delimited text format files, and the common variants in all the 3 samples were identified using VCFtools.

### Electrophoretic karyotyping.

Overnight cultures from single colonies were used as inoculum for secondary cultures, which were grown until an OD_600_ of 0.9 was reached. Cells with an OD of approximately 3 were used for chromosomal plug preparation, following the manufacturer’s instructions (Bio-Rad), using CleanCut agarose (0.6%), lyticase enzyme, and proteinase K. The chromosomes embedded in the agarose plugs were separated on a 1.0% agarose gel (Bio-Rad) using 0.5× Tris-borate-EDTA (TBE) as the running buffer. The run protocol is as follows: 60- to 60-s switch, 6 V/cm, and 120° included angle over 8 h at 12°C, followed by 90- to 150-s switch, 6 V/cm, and 120° included angle over 18 h at 12°C. The run was performed in Chef-DR III system (Bio-Rad). The gel was stained with ethidium bromide postrun, and the bands were visualized using the gel documentation system (Bio-Rad).

### CNV analysis.

The sequencing raw reads were mapped to the clade 2 genome assembly (accession no. GCA_003013715) using Yeast Mapping Analysis Pipeline (YMAP) (54). The baseline ploidy was taken as 1, C. auris being a haploid species. Corrections were applied for GC bias and chromosome end bias.

### Gene list analysis.

The lists of duplicated genes obtained from CNV analysis, and the upregulated genes obtained from RNA-seq were compared using “List operations” (https://molbiotools.com/listoperations.php) to obtain the overlapping gene sets.

### Data availability.

The raw reads of whole-genome sequencing and RNA-seq have been deposited in NCBI under BioProject accession no. PRJNA830685.

10.1128/mbio.03052-22.8 DATA SET S1 RNA-seq: differentially regulated genes in the evolved strains. Download Data Set S1, XLSX file, 0.2 MB. Copyright © 2022 Narayanan et al. 2022 Narayanan et al. https://creativecommons.org/licenses/by/4.0/ This content is distributed under the terms of the Creative Commons Attribution 4.0 International license.

10.1128/mbio.03052-22.9 DATA SET S2 Common and strain-specific SNPs identified in the evolved strains. Download Data Set S2, XLSX file, 0.02 MB. Copyright © 2022 Narayanan et al. 2022 Narayanan et al. https://creativecommons.org/licenses/by/4.0/ This content is distributed under the terms of the Creative Commons Attribution 4.0 International license.

10.1128/mbio.03052-22.10 DATA SET S3 Common duplicated and upregulated genes in F1.2 and F2.6. Download Data Set S3, XLSX file, 0.02 MB. Copyright © 2022 Narayanan et al. 2022 Narayanan et al. https://creativecommons.org/licenses/by/4.0/ This content is distributed under the terms of the Creative Commons Attribution 4.0 International license.
