# Supplementary material for: Directed Evolution Detects Supernumerary Centric Chromosomes Conferring Resistance to Azoles in Candida auris
Source: mBio. 2022 Nov 29;13(6):e03052-22. doi: 10.1128/mbio.03052-22 (PMC9765433; doi:10.1128/mbio.03052-22)
Supplement: TABLE S2 [file mbio.03052-22-s0005.docx]

**Table S2: MICs of different colonies of the evolved strains**

| Strain | Colonies | MIC (in µg/mL) |
| --- | --- | --- |
| C1 | C1.1 | 8 |
|  | C1.2 | 8 |
|  | C1.3 | 8 |
|  | C1.4 | 8 |
|  | C1.5 | 8 |
|  | C1.6 | 8 |
|  | C1.7 | 8 |
| F1 | F1.1 | 128 |
|  | F1.2 | 128 |
|  | F1.3 | 128 |
|  | F1.4 | 64 |
|  | F1.5 | 64 |
|  | F1.6 | 128 |
|  | F1.7 | >128 |
| F2 | F2.1 | <64 |
|  | F2.2 | <64 |
|  | F2.3 | 64 |
|  | F2.4 | <64 |
|  | F2.5 | <64 |
|  | F2.6 | 64 |
|  | F2.7 | 64 |
| F3 | F3.1 | 256 |
|  | F3.2 | 256 |
|  | F3.3 | 128 |
|  | F3.4 | >128 |
|  | F3.5 | 128 |
|  | F3.6 | 256 |
|  | F3.7 | 256 |
