# Supplementary figures and images for: Directed Evolution Detects Supernumerary Centric Chromosomes Conferring Resistance to Azoles in Candida auris
Source: mBio. 2022 Nov 29;13(6):e03052-22. doi: 10.1128/mbio.03052-22 (PMC9765433; doi:10.1128/mbio.03052-22)

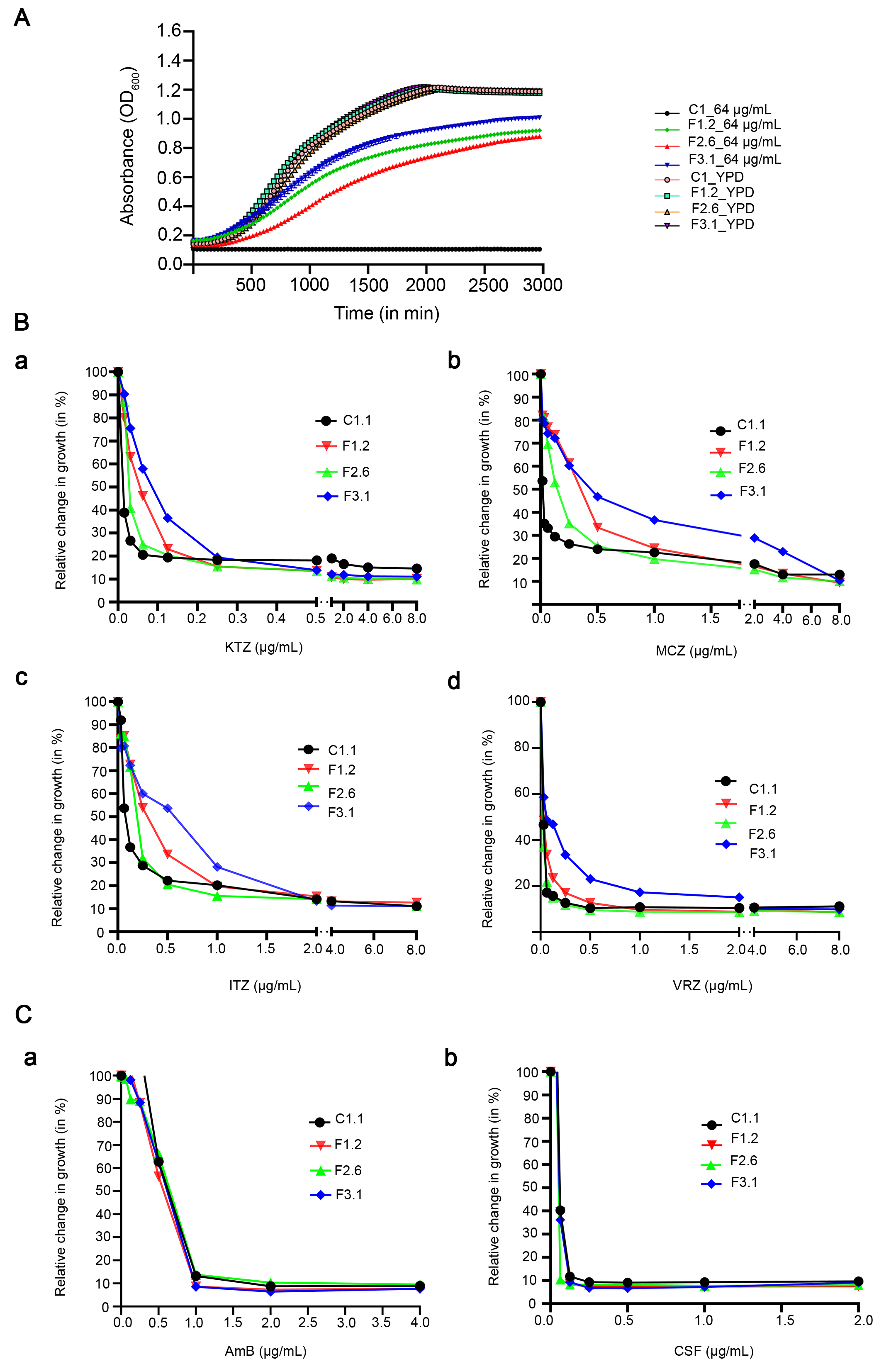

Supplement: FIG S1 [file mbio.03052-22-s0001.tif]

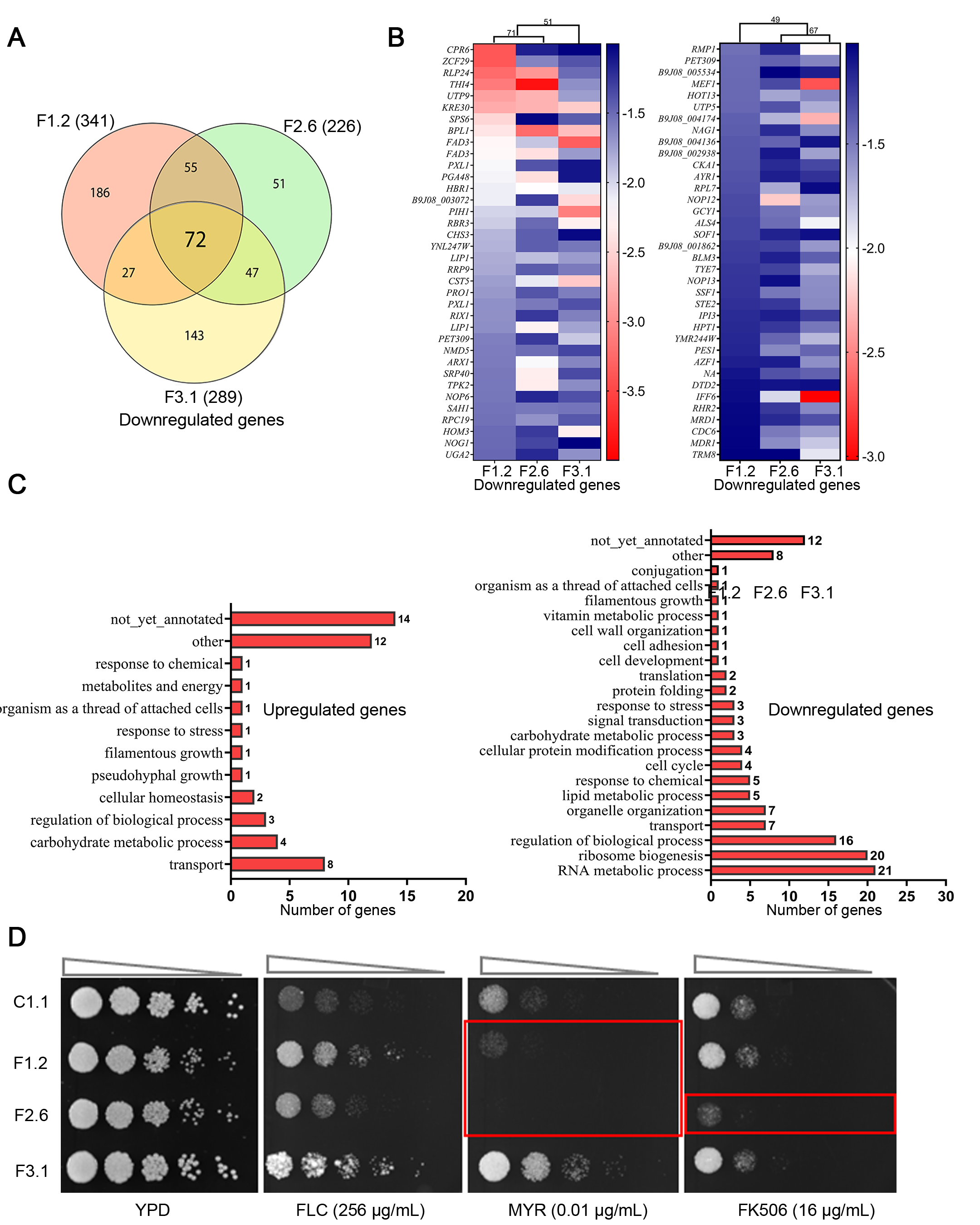

Supplement: FIG S2 [file mbio.03052-22-s0002.tif]

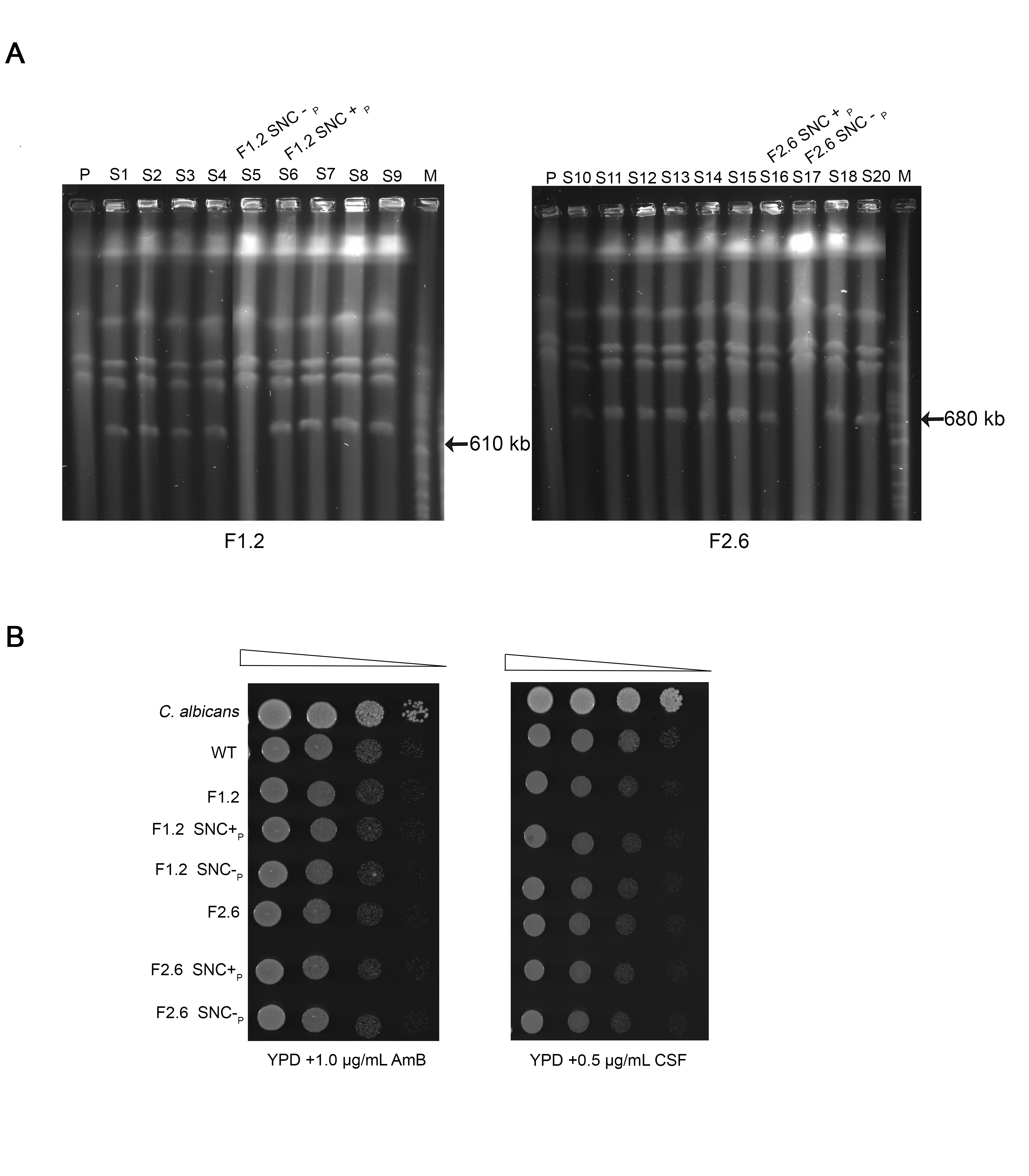

Supplement: FIG S3 [file mbio.03052-22-s0003.tif]
