# Supplementary material for: Directed Evolution Detects Supernumerary Centric Chromosomes Conferring Resistance to Azoles in Candida auris
Source: mBio. 2022 Nov 29;13(6):e03052-22. doi: 10.1128/mbio.03052-22 (PMC9765433; doi:10.1128/mbio.03052-22)
Supplement: TABLE S4 [file mbio.03052-22-s0007.docx]

**Table S4:** Putative azole-resistance associated genes in *C. auris*

| Gene-ID | Homolog in *C. albicans* | Description of *C. albicans* homolog |
| --- | --- | --- |
| B9J08_004606 | orf19.4128 | Uncharacterized |
| B9J08_004637 | No homologs listed | NA |
| B9J08_004656 | orf19.7288 | Protein with predicted oxidoreductase and dehydrogenase domains; Hap43-repressed; Spider biofilm induced |
| B9J08_004681 | orf19.6502 | Short-chain dehydrogenase/reductase; upregulation correlates with the clinical development of fluconazole resistance |
| B9J08_004770 | orf19.6770 | Protein with ENTH Epsin domain, N-terminal; Spider biofilm repressed |
| B9J08_004798 | *ARG3* | Putative ornithine carbamoyltransferase; Gcn4-regulated; Hap43-induced; repressed in the alkalinizing medium; rat catheter and Spider biofilm induced |
| B9J08_004804 | *HPD1* | 3-hydroxypropionate dehydrogenase; involved in the degradation of toxic propionyl-CoA, attenuates virulence in *C. albicans* |
| B9J08_004823 | orf19.1397 | Has domain(s) with predicted heme-binding activity |
| B9J08_004825 | orf19.1395 | Ortholog(s) have copper ion transmembrane transporter activity, inorganic phosphate transmembrane transporter activity and role in cellular copper ion homeostasis, copper ion transmembrane transport, phosphate ion transmembrane transport |
| B9J08_004828 | *GRP2* | NAD(H)-linked methylglyoxal oxidoreductase involved in the regulation of methylglyoxal and pyruvate levels; regulation associated with azole resistance; induced in core stress response or by oxidative stress via Cap1, fluphenazine, benomyl |
| B9J08_004842 | No homologs listed | NA |
| B9J08_004878 | *MEP2* | Ammonium permease and regulator of nitrogen starvation-induced filamentation; 11 predicted transmembrane regions; in low nitrogen cytoplasmic C-terminus activates Ras/cAMP and MAPK signal transduction pathways to induce filamentation |
| B9J08_004882 | orf19.5169 | Has domain(s) with predicted carbon-nitrogen ligase activity, with glutamine as amido-N-donor, hydrolase activity |

NA: Not Applicable
