# Supplementary material for: Directed Evolution Detects Supernumerary Centric Chromosomes Conferring Resistance to Azoles in Candida auris
Source: mBio. 2022 Nov 29;13(6):e03052-22. doi: 10.1128/mbio.03052-22 (PMC9765433; doi:10.1128/mbio.03052-22)
Supplement: TABLE S1 [file mbio.03052-22-s0004.docx]

**Table S1: Strains used in the study**

| **Strain** | **Description** | **Reference/Source** |
| --- | --- | --- |
| CBS10913T | Clade 2 type strain | Central Bureau voor Schimmel Cultures (CBS), Fungal Biodiversity Centre of the Royal Netherlands Academy of Arts and Sciences (KNAW), Utrecht |
| F1.2 | Strain obtained from the terminal line of the experimental evolution experiment | This study |
| F2.6 | Strain obtained from the terminal line of the experimental evolution experiment | This study |
| F3.1 | Strain obtained from the terminal line of the experimental evolution experiment | This study |
| C1.1 | Strain obtained from the control line of the experimental evolution experiment | This study |
| F1.2 SNC ^+^ _P_ | Strain obtained from the supernumerary chromosome loss experiment in F1.2 that retained the SNC | This study |
| F1.2 SNC ^-^ _P_ | Strain obtained from the supernumerary chromosome loss experiment in F1.2 that lost the SNC | This study |
| F2.6 SNC ^+^ _P_ | Strain obtained from the supernumerary chromosome loss experiment in F2.6 that retained the SNC | This study |
| F2.6 SNC ^-^ _P_ | Strain obtained from the supernumerary chromosome loss experiment in F2.6 that lost the SNC | This study |
