# Supplementary material for: Directed Evolution Detects Supernumerary Centric Chromosomes Conferring Resistance to Azoles in Candida auris
Source: mBio. 2022 Nov 29;13(6):e03052-22. doi: 10.1128/mbio.03052-22 (PMC9765433; doi:10.1128/mbio.03052-22)
Supplement: TABLE S3 [file mbio.03052-22-s0006.docx]

**Table S3:** List of primers used in this study

| **Primer name** | **Primer sequence (5’- - -3’)** |
| --- | --- |
| CAUR_00156 RT FP  CAUR_00156 RT RP | CATTCTTGGGTCTTCGTGGTGG  CCACAATCACAAGGGTCATCG |
| CAUR_01188 RT FP  CAUR_01188 RT RP | ACACCGGAAACTATTGCCACTG  ACTCTGATGGTCGCCAAAACCG |
| CAUR_00368 RT FP  CAUR_00368RT RP | GACCGCCATTGATGAAGAAAC  GCAAGGGCATTCAATAAGGA |
| CAUR_00824 RT FP  CAUR_00824 RT RP | TGGACAGAGCCAACAGACAG  ACCCAAAAGCCAAGAAACCT |
| CAUR_03761RT FP  CAUR_03761RT RP | TTCCTTGGCACGTACATTGA  GCCGTCTTCAACGACTTCTC |
| CAUR_03774RT FP  CAUR_03774RT RP | TGAATGCTTGGTGAGACTCG  TGGGGGACATCTGTATCCAT |
| CAUR_00862RT FP  CAUR_00862RT RP | AACCAAGGCTCACGAGAGAA  AACATCCAGAACGCCAAAAG |
| CAUR_00964RT FP  CAUR_00964RT RP | TCCGCTTTGAGAAGTCCAGT  CCAACGCTGAGATTGACTGA |
| CAUR_01276RT FP  CAUR_01276RT RP | GCTACGCTAGAAGCGGAGAA  TGGGGATGGTGGGTATCTAA |
| CAUR_01285RT FP  CAUR_01285RT RP | CCTGGTAAGTTGACCGCATT  GCTGAGAACACCAAGGCTTC |
| CAUR_01719RT FP  CAUR_01719RT RP | GCCTGCAAGGCTATCTTGAC  AAAGCACAACGGCAATAACC |
| CAUR_01852RT FP  CAUR_01852RT RP | GTCAACGCTCTGATTGACGA  GCGAAGTGCCAATTATCCAT |
| CAUR_02725RT FP  CAUR_02725RT RP | GGCGTCTTTGTTCAATGGTT  GAGAGGCGATGAAAGCAAAC |
| CAUR_02351RT FP  CAUR_02351RT RP | CGCTACAGTGGGGATTTGTT  TGTGTCCAACACTGGGAAGA |
| CAUR_02773RT FP  CAUR_02773RT RP | CCCTGAAGCTAATCCTGCTG  CTGCGTACGACTTGTGCCTA |
| CAUR_02951RT FP  CAUR_02951RT RP | AATGCAATTGGTTGGGATGT  AAGCAGCGTAAAAGCCGATA |
| CAUR_02994RT FP  CAUR_02994RT RP | GCATTCTTCGACGAGGCTAC  CAAGCCCTGGTGTGTACCTT |
| CAUR_03076RT FP  CAUR_03076RT RP | GGACCTAACGGTGCTGGTAA  CACTCTGTGCCCAAGTACGA |
| CAUR_03320RT FP  CAUR_03320RT RP | GGGAACACCCTTACCTGGAT  GAACGACAGCGAGAGGAAAC |
| CAUR_03795RT FP  CAUR_03795RT RP | ACCGTCACCGAAAACTTGAC  GAATTTTTCCGCATCCTTCA |
| CAUR_04133RT FP  CAUR_04133RT RP | GGAAACTTTCACGGGATTGA  TTGCCTTCTTTTCAGGCACT |
| CAUR_04233RT FP  CAUR_04233RT RP | ATGATATGGCCGTGGCTAAG  GCAGTAACGTCATCGAGCAA |
| CAUR_04562RT FP  CAUR_04562RT RP | GAGGCTCTTGCCAACAAGTC  TCAAAGCAGCACACTTACCG |
| CAUR_04813RT FP  CAUR_04813RT RP | GGAGTTGGAAACCAACCAGA  GGAAACGGAAGGAGAAGACC |
| CAUR_04824RT FP  CAUR_04824RT RP | GTGGATCAACAGCTTGAGCA  CATGCCAGCGTCTAGTTCAA |
| CAUR_04957RT FP  CAUR_04957RT RP | TGTTTGACGAGCCTTCCTCT  ATGTTGATGCCTTCCCTCAC |
| CAUR_04953RT FP  CAUR_04953RT RP | CAGGCCTGAGTTTGAAGAGG  CCAACCACCAGAAAATGCTT |
| CAUR_05555RT FP  CAUR_05555RT RP | ACAACCATCGTCGACATCAA  GCAAATAGACCATGCCGTTT |
| CAUR_02457 RT FP  CAUR_02457 RT FP | AGCTTCTGAGGGCAAATTGA  TTGGCAACGTATTCAACCAA |

FP (forward primer), RP (reverse primer), and RT (Real Time)
